# Factors affecting health and safety, particularly sharps injuries, in medical waste handlers in hospitals in the rapidly developing desert emirate of Abu-Dhabi; a questionnaire study

**DOI:** 10.1186/1753-6561-5-S6-P215

**Published:** 2011-06-29

**Authors:** NAS Helal, J Edger, L Hawkins

**Affiliations:** 1 Public Health, Postgraduate Medical School, University of Surrey, Abu-Dhabi, United Arab Emirates; 2 Public Health, Postgraduate Medical School, University of Surrey, UK; 3 Occupational Health, Research Park, Guildford, UK

## Introduction / objectives

Globally, the management of medical waste poses an increasing concern to health authorities and the public. This is particularly notable in developing countries such as the United Arab Emirates (UAE), where efforts are underway to control the problem. Objectives: to investigate the health problems facing medical waste handlers while collecting medical waste in Abu-Dhabi hospitals and to assess the effectiveness of available measures for preventing accidental sharps injuries.

## Methods

A cross-sectional survey among medical waste handlers (MWHs) was performed in five government hospitals in Abu Dhabi emirate during 2010. A sample of 250 was chosen using power and sample analysis. The data were collected using self administered questionnaires. Univariate analysis and the Chi-square test were used to analyse the data.

## Results

Of the total participants 58% were males. Over one half were aged 26-35 years and one third was aged 18-25 years. Among workers, the regular use of personal safety equipment showed marked differences. Gloves were used by 92%, face-masks by 85%, footwear 62% and goggles 42%. Of total responders; general health problems were reported in 17.4%, and "any health" in 8%. Sharps injuries were reported by 7.4%. Following an injury, all responders reported seen by a doctor. In the follow-up of 79 responders, 61% had the accident reported and filed; 16% received 3-monthly follow-ups.

## Conclusion

The results have thrown light on the many components of the collaborative aim of having a healthy, active and consequently productive workforce through a sound system of health protection of MWHs, as part of the nation's healthcare system.

## Disclosure of interest

None declared.

